# Supplementary material for: Assessing the impact of a tennis-themed sports video game on physical literacy and participation on physical activities among university students: A cluster randomized controlled trial study protocol
Source: PLoS One. 2026 Mar 16;21(3):e0343594. doi: 10.1371/journal.pone.0343594 (PMC12991259; doi:10.1371/journal.pone.0343594)
Supplement: Attachment — Submitted filename: pt-to-pt response email.docx [file pone.0343594.s002.docx]

Point-to-point response to the editorial

1. Please complete all items on the Human Participants Research Checklist that are relevant for your submission, by following this link:  <http://journals.plos.org/plosone/s/file?id=dc11/PLOSOne_Human_Subjects_Research_Checklist.docx> (Contact us at [plosone@plos.org](mailto:plosone@plos.org) if you cannot access the document.) There may be overlap between the checklist items and other queries listed below; please address any duplicated queries both in your response email and on the checklist itself. Upload the completed Human Participants Research Checklist as file type “Other” when you re-submit your manuscript. This document is for internal journal use only and will not be published if your article is accepted. The requested information will help us to assess whether your submission complies with PLOS ONE’s policies and adheres to applicable reporting standards. Note that your manuscript may be rejected if you provide incomplete or inadequate responses to the checklist questions and that changing the
   ‘Section/Category’ of your article does not affect this requirement.

RESPONSE:

The completed Human Participants Research Checklist is attached as file type ‘Other’.

2. We note that you have indicated that you received funding for your study. If you received peer reviewed external funding, your submission may be considered for expedited editorial assessment. To be considered for expedited review, please upload the Funding letter you received, and English translation, as an ‘Other’ file. Please also provide proof that the funder administered independent peer review of your protocol. Examples of evidence include: a link to a description of the funder’s peer-review process; a letter from the funding agency confirming the independent peer review process; a list of the names and credentials of the peer reviewers (if available).

Please note that we will not consider the following types of funding for expedited peer review: Funding from an author’s institution, or any other non-independent source; Funding from a commercial source; Funding that was not granted for this specific project (e.g. general PhD funding or fellowship).

If you are unable to provide proof of peer-reviewed external funding for your study, please note this in your cover letter to avoid delays in processing your manuscript. More information related to the relevant journal policies is available from: [<https://journals.plos.org/plosone/s/submission-guidelines#loc-study-protocols>].

RESPONSE:

There is no external funding for this study. We have revised the statement on page 2.

3. Please note that Study Protocols must relate to a research study that has not yet generated results. Studies that have completed either recruitment or data collection will not be considered. For further information, please see <https://journals.plos.org/plosone/s/submission-guidelines#loc-study-protocols>. Please ensure that your Methods section reports the status and timeline of the study, including an estimate of when A) participant recruitment will be completed (if applicable), B) data collection will be completed and C) results are expected. If any of these stages have already been completed, this should be indicated in the Methods section.

RESPONSE:

Thanks for the suggestions. We have included the relevant information and timeline in the Data collection section of the Methods (page 10-11)

4. We note you did not include a copy of the original, (signed or stamped) dated IRB/research ethics committee approval letter or waiver for all study sites as an ‘Other’ file. In keeping with our submission guidelines, please upload the approval letter or waiver as an ‘Other’ file. More information related to the relevant journal policies is available from: [<https://journals.plos.org/plosone/s/submission-guidelines#loc-guidelines-for-specific-study-types>]

RESPONSE:

The approval letter from the ‘Survey and Behavioural Research Ethics’ committee is attached as an ‘Other’ file.
