# Supplementary material for: Assessing the impact of a tennis-themed sports video game on physical literacy and participation on physical activities among university students: A cluster randomized controlled trial study protocol
Source: PLoS One. 2026 Mar 16;21(3):e0343594. doi: 10.1371/journal.pone.0343594 (PMC12991259; doi:10.1371/journal.pone.0343594)
Supplement: Attachment — Submitted filename: Comments.docx [file pone.0343594.s003.docx]

Summary:

The study addresses an important gap in the literature, particularly in Asian university populations, and integrates both objective and self-reported measures across multiple domains of physical literacy. The protocol is thorough, with clear inclusion/exclusion criteria, a detailed intervention plan, and ethical considerations.

Comments

The introduction is dense with literature but could benefit from clearer linkage between gaps in knowledge and the study’s specific aims.

While the sample size calculation is thorough, there is slight ambiguity regarding whether the intra-cluster correlation (ICC) was considered. Cluster designs require adjustments for ICC to avoid underestimating sample size.

Two 20-minute sessions per week may be relatively short to elicit measurable changes in physical literacy, especially in physical competence. Some rationale for the chosen duration could strengthen the protocol.

It may also be useful to clarify how variability in participant adherence (e.g., first-come-first-served access to consoles) will be handled analytically.

The tennis knowledge quiz is researcher-designed and pilot-tested but not formally validated. Consider reporting limitations and potential biases in interpretation.

Clarification on handling potential confounders, covariates, and interaction effects would strengthen the analysis plan.

The discussion section currently highlights the impact of sports video games on physical activity and literacy but lacks explicit linkage to evidence demonstrating the broader psychological and physiological benefits of structured physical activity interventions. Add below phrase to strengthen the practical relevance and theoretical grounding of the findings.

“Previous work has demonstrated that structured physical activity interventions can positively influence psychological and physiological outcomes, including mood regulation, emotional resilience, and neurochemical modulation [Nasif et al., 2025 <https://doi.org/10.31579/2578-8868/359> ]. These findings underscore the broader role of physical engagement through innovative modalities such as sports video games to promote physical literacy, enhance motivation, and support overall health among university students.”

While harms are mentioned, the likelihood of musculoskeletal or other injuries from video gaming is probably low; however, the protocol could specify how minor adverse events will be reported and categorized.

The protocol does not describe whether students in the control group are discouraged or restricted from gaming independently. This may introduce contamination bias.

Limitations Section: Acknowledge the reliance on self-reported measures and potential biases. Note that cognitive and social engagement measures are largely exploratory and context-specific.
