# Supplementary material for: Assessing the impact of a tennis-themed sports video game on physical literacy and participation on physical activities among university students: A cluster randomized controlled trial study protocol
Source: PLoS One. 2026 Mar 16;21(3):e0343594. doi: 10.1371/journal.pone.0343594 (PMC12991259; doi:10.1371/journal.pone.0343594)
Supplement: Attachment — Submitted filename: ResponseToReviewers.docx [file pone.0343594.s004.docx]

PONE-D-25-48676

Assessing the impact of a tennis-themed sports video game on physical literacy and participation on physical activities among university students: a cluster randomized controlled trial study protocol

***Journal Requirements:***

*1. Please ensure that your manuscript meets PLOS ONE's style requirements, including those for file naming. The PLOS ONE style templates can be found at*

[*https://journals.plos.org/plosone/s/file?id=wjVg/PLOSOne_formatting_sample_main_body.pdf*](https://journals.plos.org/plosone/s/file?id=wjVg/PLOSOne_formatting_sample_main_body.pdf)*and*

[*https://journals.plos.org/plosone/s/file?id=ba62/PLOSOne_formatting_sample_title_authors_affiliations.pdf*](https://track.editorialmanager.com/CL0/https:%2F%2Fjournals.plos.org%2Fplosone%2Fs%2Ffile%3Fid=ba62%2FPLOSOne_formatting_sample_title_authors_affiliations.pdf/1/010f019a0226c2ed-514fb39c-1650-4e35-882d-6ca3fa492ba8-000000/x7QKfJO1gvQCAaFDa3Wx3-Aav6sKGBbzOMAe5LYm-ZU=233)

Response: All files have been renamed and revised according to PLOS ONE’s style requirements.

*2. When completing the data availability statement of the submission form, you indicated that you will make your data available on acceptance. We strongly recommend all authors decide on a data sharing plan before acceptance, as the process can be lengthy and hold up publication timelines. Please note that, though access restrictions are acceptable now, your entire data will need to be made freely accessible if your manuscript is accepted for publication. This policy applies to all data except where public deposition would breach compliance with the protocol approved by your research ethics board. If you are unable to adhere to our open data policy, please kindly revise your statement to explain your reasoning and we will seek the editor's input on an exemption. Please be assured that, once you have provided your new statement, the assessment of your exemption will not hold up the peer review process.*

Response: The Data Availability Statement has been revised:

“The datasets generated and/or analyzed during the current study will be made publicly available upon acceptance of the manuscript. Data will be deposited in the Open Science Framework (OSF) repository (https://osf.io), and a DOI or permanent link will be provided at the time of publication. If any portion of the data is subject to ethical restrictions based on the protocol approved by the research ethics board, access will be limited accordingly, and a justification will be included.” (line 39-45)

*3. Your ethics statement should only appear in the Methods section of your manuscript. If your ethics statement is written in any section besides the Methods, please move it to the Methods section and delete it from any other section. Please ensure that your ethics statement is included in your manuscript, as the ethics statement entered into the online submission form will not be published alongside your manuscript.*

Response: The ethics statement has been moved exclusively to the **Methods** section of the manuscript. Any duplicate mentions in other sections have been removed.

*4. If the reviewer comments include a recommendation to cite specific previously published works, please review and evaluate these publications to determine whether they are relevant and should be cited. There is no requirement to cite these works unless the editor has indicated otherwise.*

Response: We have carefully reviewed the publications suggested by the reviewers. Relevant citations have been incorporated into the manuscript where appropriate.

***Point-to-point Responses to Reviewers***

***Reviewer #1: Summary:***
*The study addresses an important gap in the literature, particularly in Asian university populations, and integrates both objective and self-reported measures across multiple domains of physical literacy. The protocol is thorough, with clear inclusion/exclusion criteria, a detailed intervention plan, and ethical considerations.

Comments
1. The introduction is dense with literature but could benefit from clearer linkage between gaps in knowledge and the study’s specific aims.*
Response: Thanks for your suggestion. We have added this linkage in the introduction section. The revised text now reads:

“While existing research has largely focused on the affective and cognitive benefits of SVGs, there remains a notable gap in studies exploring their influence on physical competence and the social domains of PL [16]. …These gaps underscore the need for interventions addressing all PL domains rather than focusing narrowly on motivation or knowledge.” (line 207-209)

“…Therefore, the aim of this study is to investigate the effectiveness of a tennis-themed SVG intervention, implemented alongside regular physical education lessons, in promoting holistic PL among university students in Hong Kong. Specifically, the study will evaluate whether SVG participation can enhance motivation, sport-specific knowledge, physical competence, social engagement, and physical activity engagement. Using a cluster randomized controlled trial design, this research seeks to identify innovative, technology-enhanced strategies to improve PE outcomes and foster lifelong physical activity.” (line 261-268)

*2. While the sample size calculation is thorough, there is slight ambiguity regarding whether the intra-cluster correlation (ICC) was considered. Cluster designs require adjustments for ICC to avoid underestimating sample size.*
Response: Thank you for highlighting this important point. To account for the ICC to avoid underestimating the required sample size, we applied the design effect formula. The target samples size has been adjusted. (line 349-364)

*3. Two 20-minute sessions per week may be relatively short to elicit measurable changes in physical literacy, especially in physical competence. Some rationale for the chosen duration could strengthen the protocol.*

Response: Thank you for your valuable suggestion. We have incorporated a relevant reference to support the intervention design in the revised manuscript. Specifically, we added the following paragraph to the Study Design section:

“The intervention duration of two 20-minute sessions per week was selected based on feasibility within the university schedule and supported by prior research. Röglin et al. [17] conducted a randomized controlled trial in which 15–20-minute exergaming sessions, delivered twice weekly over three months, significantly improved students’ physical self-concept, including strength, coordination, and speed. These findings suggest that even short-duration, structured gaming interventions can elicit measurable changes in physical competence and support physical literacy development.” (line 317-324)

*4. It may also be useful to clarify how variability in participant adherence (e.g., first-come-first-served access to consoles) will be handled analytically.*

Response: We have clarified this aspect in the part of Intervention in the revised manuscript:

“…Due to equipment limitations (2 consoles with a total of 8 controllers), each session has limited quota. If a session reaches its quota due to the first-come-first-served arrangement, we will arrange an alternative time slot to accommodate affected participants. The attendance will be tracked weekly, in case where participants are unable to fulfill the attendance requirement, we will contact them via email to arrange make-up sessions. This ensures that all participants can fulfill the attendance requirement and receive the full intended dosage of the intervention.” (line 432-439)

*5. The tennis knowledge quiz is researcher-designed and pilot-tested but not formally validated. Consider reporting limitations and potential biases in interpretation.*

Response: Thank you for your helpful comment regarding the tennis knowledge quiz. This is an important limitation, and we have addressed it in the revised manuscript.

“…The tennis knowledge quiz used in this study was developed by the research team and pilot-tested for clarity and relevance. However, it has not been formally validated, which may introduce measurement bias and limit the generalizability of findings related to cognitive domain outcomes. Future studies should consider using or developing standardized, validated instruments to assess sport-specific knowledge.” (line 708-713)

*6. Clarification on handling potential confounders, covariates, and interaction effects would strengthen the analysis plan.*
Response: Thanks for your suggestion. We have added this part in the Data Analysis:

“To strengthen the validity of our findings, we will account for potential confounders, covariates, and interaction effects in our statistical analysis. Confounding variables such as baseline physical activity levels, prior experience with sports video games, and demographic characteristics (e.g., age and gender) will be identified and adjusted for in the regression models. In particular, prior SVG experience will be treated as a covariate, as it may influence participants’ engagement with the intervention and their baseline competencies in reaction time and eye-hand coordination. Additional covariates including self-reported motivation for physical activity and baseline physical literacy scores will be incorporated to improve model precision and control for individual variability. Furthermore, we will explore interaction effects between the intervention and participant characteristics, such as gender and gaming experience, to determine whether the intervention’s impact varies across subgroups. These analytical strategies will enhance the precision of our findings and provide a clearer understanding of the intervention’s impact across diverse participant profiles.” (line 649-662)

*7. The discussion section currently highlights the impact of sports video games on physical activity and literacy but lacks explicit linkage to evidence demonstrating the broader psychological and physiological benefits of structured physical activity interventions. Add below phrase to strengthen the practical relevance and theoretical grounding of the findings.
“Previous work has demonstrated that structured physical activity interventions can positively influence psychological and physiological outcomes, including mood regulation, emotional resilience, and neurochemical modulation [Nasif et al., 2025* [*https://doi.org/10.31579/2578-8868/359*](https://doi.org/10.31579/2578-8868/359) *]. These findings underscore the broader role of physical engagement through innovative modalities such as sports video games to promote physical literacy, enhance motivation, and support overall health among university students.”*

Response: Thank you for pointing this out and for the suggestion. The above phrase has been added into the Introduction section. (line 174-179)

*8. While harms are mentioned, the likelihood of musculoskeletal or other injuries from video gaming is probably low; however, the protocol could specify how minor adverse events will be reported and categorized.*
Response: Thanks for the suggestion. We have revised the ‘Harm’ section. The updated text now read:
‘…Although the likelihood of musculoskeletal or other injuries from sports video gaming is low, minor adverse events such as discomfort, fatigue, or strain may still occur. In particular, physical testing procedures such as the Alternate Hand Ball Toss Test may pose a slight risk of upper limb strain or coordination-related discomfort due to repetitive movements. All minor adverse events will be categorized by type, severity, and relation to either the intervention or testing activity, and reviewed regularly by the research team to ensure participant safety and transparency throughout the study.” (line 792-799)

*9. The protocol does not describe whether students in the control group are discouraged or restricted from gaming independently. This may introduce contamination bias.*
Response: Thanks for pointing this out. We added this part into the Intervention section, the revised text now reads:

“To minimize contamination bias, students in the control group will not be provided access to the SVG Corner and will be advised not to engage in tennis-themed sports video gaming independently during the study period. While we cannot fully restrict external gaming behavior, participants will be informed of the importance of maintaining their assigned condition and asked to report any SVG-related activity outside the study. This will help us monitor potential crossover and account for it in the final analysis.” (line 440-446)

*10. Limitations Section: Acknowledge the reliance on self-reported measures and potential biases. Note that cognitive and social engagement measures are largely exploratory and context-specific.*

Response: Thank you for your suggestion. We have added a discussion section that includes the stated limitations. (line 684-726)

***Reviewer #2:***

*1. In abstract there is no explicit research question or hypothesis mentioned upon which papers usually based. It can be seen that more focus on procedural detail is given (e.g., “two 20-minute sessions per week for six weeks”); this level of detail belongs in Methods, not the abstract. One more thing can be seen as more focus is put on general background (sales data, gaming popularity) rather than specific causal mechanisms. There are some repetition of phrases present such as “motivational and cognitive aspects,” “complementing PE lessons,” which dilute precision.*

Response:

Thank you for your constructive feedback. In the revised abstract, we have stated the study’s aim to strengthen its focus (line 63-66). Methodological specifics are more concise and placed the details in the Methods section. (line 71-75)

The specific mechanism by which sports video games enhance real-life sports involvement has been incorporated into the Introduction section. The revised text now reads:

“… According to Adachi and Willoughby [10], playing SVGs can foster a sense of competence and mastery, which boosts self-confidence and encourages involvement in physical sports. SVGs also simulate sports environments and strategies, increasing cognitive familiarity and reducing barriers to real-world participation…” (line 189-193)

Repetitive phrases like “motivational and cognitive aspects” and “complementing PE lessons” have streamlined to improve precision and clarity.

*2. There is one thing that can be observed which is lack of explanation of why certain PL measures (e.g., APLQ subset) were selected over full validated scales. There are no mention of limitations or risk nor any discussion took place in the manuscript. (e.g., Hawthorne effect, self-report bias). Also, lacks “theoretical contribution” narrative—merely describes procedures present that can be improved upon.*

Response: Thanks for the comments. We have added the rationale for choosing subset of APLQ in the Method section, and mention for limitations in Discussion section. The revised text now reads:

“iv) Social engagement in physical activities

To assess the social dimension of PL…It was selected because it is the **only available validated tool that explicitly includes the social domain**, which was central to this study’s objectives…” (line 554-556)

Discussion:

“Measurement-related constraints also warrant consideration. The study relies on self-reported measures for physical activity and gaming behavior, which are subject to recall bias and social desirability bias and may affect data accuracy. Moreover, participants’ awareness of being observed during the intervention may lead to the **Hawthorne effect**, where behavior changes occur simply because of observation rather than the intervention itself. These factors should be considered when interpreting the findings. Performance-based assessments can carry measurement error and may not fully capture the breadth of physical literacy in authentic contexts. The reliance on self-reported measures introduces potential biases that should be interpreted with caution…” (line 700-708)

A section of Theoretical contribution has been added in Discussion section.

“Theoretical Contribution

This work aims to extend physical literacy theory by exploring its application within technology-enhanced learning environments. Current frameworks primarily emphasize traditional pedagogical approaches, with limited consideration of digital tools. By incorporating SVG into structured PE lessons, this study seeks to demonstrate how interactive technologies can operationalize all four domains of physical literacy: physical, cognitive, affective, and social, within a hybrid learning context. The inclusion of the social domain, assessed through the APLQ subset, addresses a notable gap in existing literature where social engagement is often underrepresented. These insights are expected to inform conceptual models that position digital interactivity as a legitimate pathway for physical literacy development.” (line 728-738)

*3. Author can also take example such as - “AI-assisted interactive systems have demonstrated increased engagement and comprehension in technology-mediated environments, supporting the educational potential of game-based interventions.” DOI - doi.org/10.47363/JAICC/2024(3)380*
Response:

Thanks for your suggestion. We have revised the Introduction Section. The revised texts now reads:

“Traditional physical education (PE) programs have primarily focused on developing motor skills and fitness, often overlooking opportunities to integrate technology-driven approaches that can enhance engagement and learning. Recent advancements in educational technology have opened new pathways for promoting PL through interactive and immersive experiences. **AI-assisted interactive systems have demonstrated increased engagement and comprehension in technology-mediated environments, supporting the educational potential of game-based interventions [5].** These systems leverage adaptive feedback and personalized learning to create dynamic environments that foster motivation and skill acquisition. In the context of PE, sports video gaming (SVG) represents a promising application of these principles, offering students an engaging platform to practice decision-making, strategy, and collaboration while reinforcing physical skills.” (line 162-173)

*4. Authors can explain and expand cultural adaptation steps (translation, back-translation, pilot testing). There is also scope of including mixed-model analysis (random effects for clusters), report partial eta-squared and confidence intervals for primary outcomes.*

Response:

Thank you for your valuable feedback. We appreciate the suggestion regarding cultural adaptation procedures. Due to limited manpower and resources, we regret that we are unable to conduct a full cultural adaptation process at this stage. However, we have ensured that the existing instruments are appropriate for our target population based on prior use in similar contexts and expert review.

Regarding the statistical analysis, we have updated the Data Analysis plan to include mixed-effects models, and reporting partial eta-squared and CI for primary outcomes. The revised text now reads:

“To further account for the hierarchical structure of the data and potential intra-cluster correlation among participants within the same class group, a **mixed-effects model with random effects for clusters** (i.e., class groups) will be employed. This model will include fixed effects to estimate the impact of the intervention and relevant covariates, while random effects will capture variability across different class groups. This approach ensures more accurate standard error estimation and valid statistical inference by adjusting for the non-independence of observations within clusters.

To enhance the interpretability and transparency of our findings, **partial eta-squared** will be reported for all primary outcomes to indicate the magnitude of observed effects. Additionally, **95% confidence intervals** will be provided to reflect the precision of the estimates and the range within which the true effect is likely to fall. These metrics will support a more nuanced understanding of the intervention’s impact and facilitate comparison with other studies.” (line 663-675)
