# Supplementary material for: Assessing the impact of a tennis-themed sports video game on physical literacy and participation on physical activities among university students: A cluster randomized controlled trial study protocol
Source: PLoS One. 2026 Mar 16;21(3):e0343594. doi: 10.1371/journal.pone.0343594 (PMC12991259; doi:10.1371/journal.pone.0343594)
Supplement: Attachment — Submitted filename: Response To Reviewers.docx [file pone.0343594.s005.docx]

PONE-D-25-48676R1
Assessing the impact of a tennis-themed sports video game on physical literacy and participation on physical activities among university students: a cluster randomized controlled trial study protocol

**Journal Requirements:**

*1. If the reviewer comments include a recommendation to cite specific previously published works, please review and evaluate these publications to determine whether they are relevant and should be cited. There is no requirement to cite these works unless the editor has indicated otherwise.* 
Response: We have carefully reviewed the publications suggested by the reviewers. Relevant citations have been incorporated into the manuscript where appropriate.

*2. Please review your reference list to ensure that it is complete and correct. If you have cited papers that have been retracted, please include the rationale for doing so in the manuscript text, or remove these references and replace them with relevant current references. Any changes to the reference list should be mentioned in the rebuttal letter that accompanies your revised manuscript. If you need to cite a retracted article, indicate the article’s retracted status in the References list and also include a citation and full reference for the retraction notice.*
Response: We have further reviewed the reference list to ensure completeness and accuracy. All entries have been checked for correct details, and no retraction records were found.

**Additional Editor Comments:**

There are some minor changes required which have been raised by the reviewer. After that manuscript is acceptable to publish.

**Reviewer #2:**

*1. Abstract subtly overextends its claims through implication rather than direct assertion. The inclusion of objective measures such as reaction time and eye–hand coordination suggests physical competence enhancement, yet the abstract does not explicitly articulate how a sedentary gaming intervention is expected to influence these outcomes.*
Response: Thank you for your comment. We have revised the abstract to articulate this rationale. The text now reads:

“…Although the intervention is sedentary, SVG gameplay often requires reflexes, eye–hand coordination, and cooperative strategies, which may influence aspects of physical competence such as reaction time and coordination…” (line 62-64)

*2. Introduction adopts a relatively uncritical stance toward the physical literacy construct itself. While the authors accurately describe PL as multidimensional, they do not sufficiently acknowledge ongoing theoretical debates regarding its measurement and operationalization.*
Response: Thank you for highlight this important point. To address this, we have revised the introduction to include a discussion of these debates. The section now reads:

“…While physical literacy is widely accepted as a guiding framework, ongoing theoretical debates persist regarding its measurement and operationalization. For example, Boldovskaia et al. [5] emphasize that despite PL’s conceptual acceptance, there is considerable debate over appropriate methods for collecting empirical data, with most existing instruments focusing narrowly on certain dimensions.” (line 103-107)

*3. The hypotheses related to physical competence warrant closer scrutiny. The manuscript treats improvements in reaction time and eye–hand coordination as indicators of enhanced physical competence attributable to the intervention. This framing risks conflating perceptual–cognitive improvements with embodied motor competence.*
Response: Thank you for raising this important point. We have revised the manuscript to clarify this distinction in the introduction, the hypothesis and the limitation sections.

“…Moreover, gameplay often involves perceptual-motor demands such as reflexes and coordination and incorporates cooperative or competitive social interactions. These considerations underscore the need for interventions addressing all PL domains rather than focusing narrowly on motivation or knowledge.

In this study, simple reaction time and eye–hand coordination were selected as outcome measures because they represent targeted components within the physical domain of PL and align with the perceptual–motor demands of both video gaming and real-life tennis. While these measures do not capture comprehensive motor competence, they provide insight into specific physical attributes that may be influenced by the intervention.” (line 161-175)

“***Physical Competence:*** Participants in the intervention group will demonstrate greater improvement in perceptual-motor skills, specifically simple reaction time and eye-hand coordination, compared to those in the control group. These measures represent targeted components within the physical domain of PL and align with the perceptual-motor demands of both sports video gameplay and real-life tennis, rather than comprehensive motor competence.” (line 227-235)

“…Specifically, the study assessed selected perceptual-motor components (simple reaction time and eye-hand coordination) rather than comprehensive motor competence, which limits interpretation of physical literacy development to specific attributes…” (line 739-743)

*4. Although the manuscript notes that courses follow standardized content, variability in teaching style, enthusiasm, and informal reinforcement of the intervention is inevitable in real-world PE settings. While this does not invalidate the design, it should be more explicitly acknowledged and analytically addressed.*

Response: Thanks for the suggestion. We have revised the Data Analysis Sections:

“To evaluate intervention effects, mixed-effects models will be employed instead of simple repeated-measures ANOVAs to account for the hierarchical structure of the data and potential intra-cluster correlation among participants within the same class group. This approach addresses the reality that, although courses follow standardized content, variability in teaching style, instructor enthusiasm, and informal reinforcement of the intervention is inevitable in real-world PE settings. Mixed-effects models will include fixed effects for time (baseline, post-intervention, follow-up) and group (intervention vs. control), as well as random effects for class clusters to capture variability across different teaching contexts. These models will assess both main effects and interaction effects, providing more accurate standard error estimation and valid statistical inference.” (line 604-613)

*5. What is less developed is the explicit articulation of the intervention’s theoretical mechanism. While the manuscript describes what participants do during gameplay, it stops short of clearly mapping specific game mechanics to expected cognitive, affective, or behavioral outcomes.*

Response: Thanks for pointing this out. We have added the game mechanics in the introduction section:

“The intervention leverages specific game mechanics to target physical literacy domains and foster motivation for real-life tennis participation rather than increased video gaming. Fast-paced rally sequences and timing-based challenges aim to improve perceptual-motor skills such as reaction time and eye-hand coordination (physical domain). In-game tutorials and rule-based decision-making tasks support sport-specific knowledge (cognitive domain). Immersive graphics, realistic physics, and authentic match scenarios are designed to enhance enjoyment and create a sense of competence, which may translate into motivation to engage in actual tennis (affective domain). Multiplayer modes encourage peer interaction and collaboration (behavioral domain). Together, these mechanics are intended to promote multidimensional physical literacy development and bridge virtual engagement with real-world physical activity.” (line 194-204)

**Reviewer #3:**

*1. Authors have done well job on revising their manuscript. I think manuscript has improved in a great level.*

Response: Thank you very much for your positive feedback and for recognizing the improvements made to the manuscript. We greatly appreciate your time and effort in reviewing our work and providing constructive comments that helped us enhance the quality of the paper.
